# Evaluation of Analytical Modeling Functions for the Phonation Onset Process

**DOI:** 10.1155/2016/8469139

**Published:** 2016-03-15

**Authors:** Simon Petermann, Stefan Kniesburges, Anke Ziethe, Anne Schützenberger, Michael Döllinger

**Affiliations:** Division of Phoniatrics and Pediatric Audiology at the Department of Otorhinolaryngology Head & Neck Surgery, University Hospital Erlangen, Medical School, Friedrich-Alexander University Erlangen-Nürnberg (FAU), Bohlenplatz 21, 91054 Erlangen, Germany

## Abstract

The human voice originates from oscillations of the vocal folds in the larynx. The duration of the voice onset (VO), called the voice onset time (VOT), is currently under investigation as a clinical indicator for correct laryngeal functionality. Different analytical approaches for computing the VOT based on endoscopic imaging were compared to determine the most reliable method to quantify automatically the transient vocal fold oscillations during VO. Transnasal endoscopic imaging in combination with a high-speed camera (8000 fps) was applied to visualize the phonation onset process. Two different definitions of VO interval were investigated. Six analytical functions were tested that approximate the envelope of the filtered or unfiltered glottal area waveform (GAW) during phonation onset. A total of 126 recordings from nine healthy males and 210 recordings from 15 healthy females were evaluated. Three criteria were analyzed to determine the most appropriate computation approach: (1) reliability of the fit function for a correct approximation of VO; (2) consistency represented by the standard deviation of VOT; and (3) accuracy of the approximation of VO. The results suggest the computation of VOT by a fourth-order polynomial approximation in the interval between 32.2 and 67.8% of the saturation amplitude of the filtered GAW.

## 1. Introduction

The voice is an essential part of human communication and in modern times has become increasingly important in professional and private life. As voice-based communication increases, however, voice disorders are more frequently encountered and financial costs increase [1]. Particularly in professions such as teaching, there is often an overuse of voice, which significantly increases the prevalence of voice disorders [2]. A better understanding of the complex process of vocalization is essential for new and more effective treatments of persons suffering voice disorders or even loss of voice.

**Figure 1:**
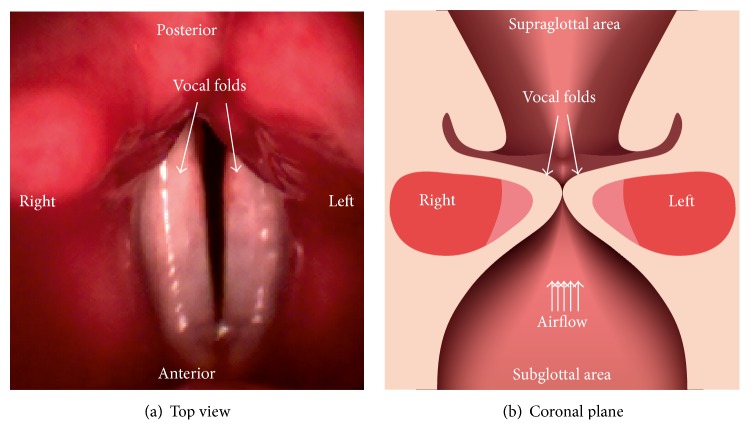
(a) Top view of vocal folds; (b) view of the coronal plane of the larynx with subglottal area, vocal folds, and supraglottal area depicted.

The primary voice signal originates from the vibrating vocal folds [3]. Subsequently, this primary voice signal is modulated in the vocal tract, generating the normal voice signal. The beginning of vocalization is denoted as the voice onset (VO). This is the event after the vocal folds have adducted and the air starts to flow from the lungs through the larynx and the vocal folds are initiated to vibrate (Figure 1) [4]. Thus, VO is the transition from damped to sustained vocal fold oscillations [5].

There are different ways to observe the vocal fold dynamics (100–400 Hz during normal phonation) directly. Currently used visualization techniques are videostroboscopy, high-speed videokymography (HSK), and high-speed videoendoscopy (HSE) [6]. Videostroboscopy is widely applied for clinical examinations of vocal fold vibrations and represents the gold standard. However, videostroboscopy is not suitable for observing irregular or nonperiodic oscillations in pathological voices or the VO [6]. In contrast, HSK uses higher frame rates of up to 8000 fps and hence is suitable for examining the entire phonation cycle and recording irregularities within the oscillations. However, HSK shows the vocal folds dynamics just at a single horizontal line across the vocal folds (i.e., one trajectory at one vocal fold position) and therefore does not reflect the entire vocal fold vibrations. In contrast, HSE enables the entire superior vocal fold dynamics to be visualized. To visualize and analyze the vocal fold oscillations from the HSE recordings, the area of the glottis is segmented for each frame [7]. By stringing together the glottal area of each frame, the glottal area waveform (GAW) is generated, reflecting the vocal fold vibrations [8].

Moreover, owing to increasing performance of digital high-speed camera chips, it is possible to use flexible nasally introduced endoscopes instead of the commonly used rigid endoscopes for HSE at high frame rates of up to 20000 fps [9]. The flexible endoscope affects the phonatory process less because it is nasally induced and therefore does not restrict the flexibility of the tongue as much as a rigid endoscope. Only by the flexible endoscope, the vocal fold oscillations can be visually inspected during articulation, for example, the disyllabic word [‘mama] being analyzed in our study.

The VO as a transient effect with its short irregular oscillations of the vocal folds may contain valuable information for assessing the vocal fold characteristics that determine vibratory function [6]. By analyzing the nonstationary VO, pathological voices can be differentiated from healthy voices [10]. Also, age-related changes in vocalization, which become increasingly important in a society with an increasing life span, are measurable during VO [6].

In previous studies, VO was analyzed based on acoustic, electroglottographic (EGG), and HSE signals (trajectories and GAW) in addition to aerodynamic measures such as the phonation threshold pressure [6, 10–15]. VO characteristics have been analyzed in in vivo [6, 11, 14] and in vitro studies based on physical models [16] or excised human larynges [15].

Different measures such as the glottal parameter open quotient [11, 17] and the duration of the voice onset (voice onset time, VOT) have been derived from HSE signals to quantify the VO [10, 13, 17]. The VOT especially seems to be a promising measure.

For HSE signals, VOT has been determined by trajectories [5, 10] or GAWs [18], on filtered [5, 10] and unfiltered signals [18]. Kunduk et al. [18] fitted a polynomial function to the peaks of the GAW. Mergell et al. [5] fitted an analytic envelope curve based on the analysis of the Hopf bifurcation representing the dynamic system at the onset of oscillation (transition from damped to sustained vocal fold oscillations) to the Hilbert envelope of the trajectory function. Also, the definition of the VOT referring to the full opening of the glottis differs. Definitions of the interval of VO were set from 5 to 90% (VOT_90_) [18] and from 32.2 to 67.8% (VOT_67_) [5, 10] of the saturation amplitude [17].

These different approaches and definitions of the VOT show that there is no standardized determination or computation of the onset process yet. Therefore, it is difficult to compare the results of different studies carried out to determine VOT. In the various studies analyzing the VO using different methods, there is wide intersubject and intrasubject variability within and between the different studies [11]. Variability of the results concerning the VOT might be partially due to the methods used to determine VOT.

Thus, and owing to the advantages of HSE recordings in combination with GAW analysis as described above, we sought to investigate which method might be the most robust and reliable way to quantify automatically and objectively the VOT from the GAW. Hence we investigated different existing methods to determine the VOT based on the GAW. In particular we analyzed the function *M*(*t*) [5] with a different quantity of parameters being optimized (*M*_*a*_(*t*), *M*_*as*_(*t*), and *M*_*asr*_(*t*)) and polynomials [18] of second *P*_2_(*t*), third *P*_3_(*t*), and fourth order *P*_4_(*t*). Additionally, we combined different parts of the methods to find the potentially best combination of fit functions. Furthermore, we applied them on filtered or unfiltered GAW and with different definitions of the VOT referring to the percentage of the saturation amplitude on which the computation of the VOT is based. The goal was to determine the potentially best combination of raw data, fit function, and calculation rule for VOT to find a robust and reliable procedure for determining the VOT based on the GAW. Besides, we aimed to suggest improvements to the existing methods.

A further methodological novelty in this study is that we used HSE recordings at 8000 fps with a flexible endoscope for the analysis of VO. The flexible and nasally inserted endoscope has less effect on the vocalization or speech production process; hence in the analysis of the VOs this allows articulations or words to be closer to normal voice use than just phonating a vowel.

## 2. Method

### 2.1. Subjects

Nine men (age 23.9 ± 2.7 years) and 15 women (age 24.4 ± 2.2 years) participated in the study. All of the subjects were native German speakers. No voice or hearing impairments were diagnosed in the pretest examination. None of the subjects were trained singers and all of them signed a consent form. The experiments conformed to the Declaration of Helsinki (1964) and were approved by the local ethical committee (approval number 4364).

### 2.2. Test Setup

**Figure 2:**
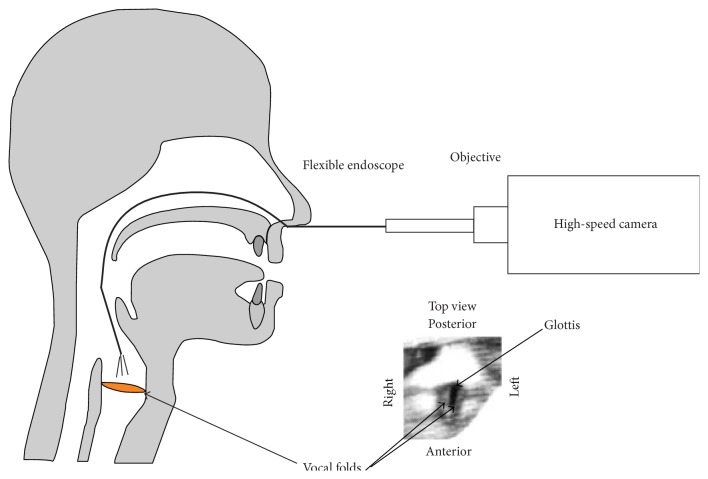
Schematic illustration of transnasal high-speed endoscopy with a flexible endoscope. Bottom right: top view of the vocal folds and glottis as seen through the camera; figure after image enhancement (Section 2.3).

The subjects heard the word [‘mama] spoken by a model speaker over headphones to memorize speed of pronunciation and intonation. The subjects articulated [‘mama] at a convenient loudness level at around 75 dB. Each subject produced around 20 [‘mama]s, which were recorded. Simultaneously, the vocal fold vibrations were recorded with a Photron SA1.1 high-speed (hs) camera coupled to a flexible fiber endoscope (Olympus) via a 25 mm Storz objective. The endoscope was introduced nasally (Figure 2). The spatial resolution of the hs recordings was 128 × 128 pixels at a frame rate of 8000 fps. A 270 W Storz light source was attached to the flexible endoscope to illuminate the vocal folds during the recordings. Depending on the video (i.e., visibility of the entire vocal folds) and the segmentation quality (see Section 2.4), some videos were excluded from further analysis. Overall, 126 recordings of the nine male subjects and 210 recordings of the 15 female subjects were evaluated.

### 2.3. Image Processing

**Figure 3:**
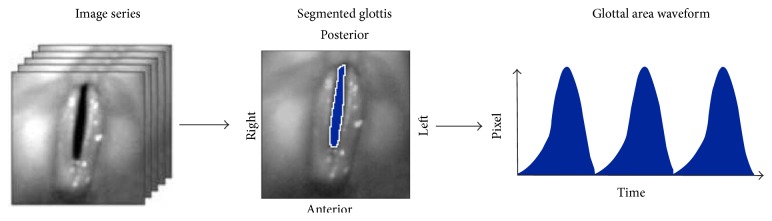
Left: image series of hs recordings of vocal folds; middle: single hs image of vocal folds with segmented glottis—blue area; right: function of the glottal area waveform (GAW) derived from the segmented image series over time (i.e., image series). Maxima in the GAW correspond to an open glottis and zero values to a closed glottis.

Image processing was conducted to improve the image quality in terms of brightness and contrast and to allow for a more accurate segmentation of the hs recordings (Figure 3). A 50 Hz flickering induced by the light source and interferences due to fractionation at the fiber optic impaired the quality of the hs recordings. Therefore, three steps of image processing were conducted. A stretching of the grey-scale values to the whole grey scale of 256 values to increase brightness and to reduce temporal variations in brightness induced by the 50 Hz flickering of the light source was performed. A spatial low-pass filter with a linear response was applied to reduce the interference patterns. Finally, clipping off the upper 50% of the grey-scale range and distributing the remaining grey-scale values over the whole grey-scale range were conducted, again to increase the contrast between the dark glottis and the surrounding vocal folds.

### 2.4. Glottal Area Waveform (GAW)

**Figure 4:**
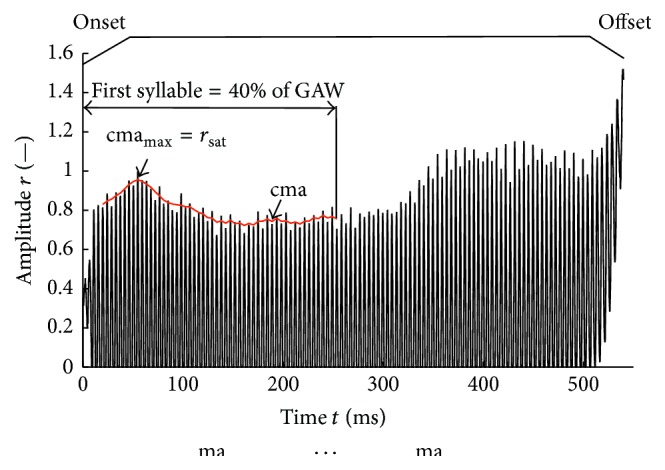
Normalized glottal area waveform of the word [‘mama] representing a female subject. Onset, offset, and central moving average cma (red) over normalized first syllable and saturation amplitude *r*_sat_ are depicted. The cma (red) starts at the fifth peak because (1) the first two peaks are excluded from cma calculation because there is no glottal closure before (Section 2.6) and (2) the kernel size of the cma is five.

The glottal area was determined via the in-house segmentation tool “Glottis Analysis Tools (GAT)” for each frame. The glottal area waveform (GAW) was then generated automatically. The GAW is the glottal area in pixel units over time (Figure 3). In the following the GAW is shown in diagrams of the normalized glottal area *r* as function of the time *t*. Therein, the GAW was normalized to the maximum peak of the first syllable of the word [‘mama] (Figure 4).

### 2.5. Definition of the Voice Onset

*Voice Onset*. For our purposes, the phonation process can be divided roughly into three major parts (Figure 4). The first is the phonation onset, that is, the event when the tracheal air flow from the lungs starts to pass through the adducted vocal folds, which begin to vibrate. During this process, the oscillation amplitude of the vocal folds increases until they reach their maximal amplitudes and pass on to a periodic oscillation state. This periodic state is called sustained phonation. When ending the phonation (i.e., sustained state), the amplitudes decrease until the vocal fold oscillations stop entirely. This process is called the phonation offset. The duration of onset or offset is then consequently denoted as the phonation/voice onset time (VOT) and voice offset time. In our study, we focused only on VOT. At the very beginning of the voice onset and during the offset, the vocal folds oscillate but do not touch each other (Figure 4).

*Saturation Amplitude*. *r*_sat_ is defined as the mean oscillation amplitude during sustained phonation obtained from the GAW. As the GAW of an articulation of a word does not show constant amplitudes, the saturation amplitude was defined as the maximum of the central moving average of the peak values cma_max_ of each oscillation cycle with a kernel size of five peaks during the first syllable, which corresponds to the first 40% of the GAW (Figure 4).

*Voice Onset Time (VOT)*. The VOT was determined based on analytical functions (Table 1 and (1)) that model the peak devolution of the GAW. The functions were fit over all peaks between the first fitting point (see Section 2.6) and the last peak included in the cma_max_ computation. We tested two different intervals, given in the literature, for computing the VOT.

**Figure 5:**
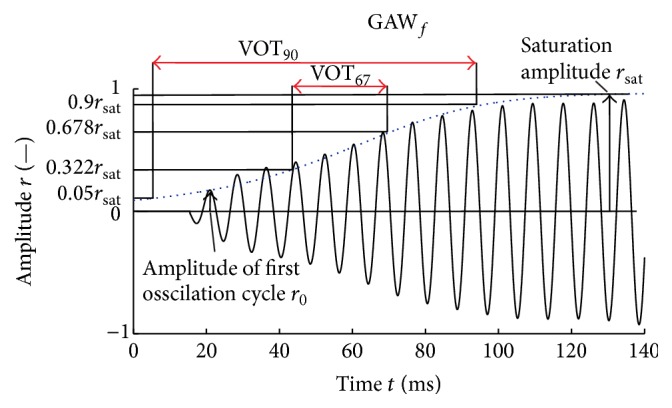
Applied analytical fit function (dotted blue line) approximating the envelope of a male subject's filtered GAW. Boundaries of VOT_67_ and VOT_90_, amplitude of first oscillation cycle *r*_0_, and saturation amplitude *r*_sat_ are depicted.

*VOT*_*67*_. VOT_67_ is defined as the time period that the fit function needs to reach 67.8% of the saturation amplitude *r*_sat_ starting from 32.2% of the saturation amplitude [5] (Figure 5).

*VOT*_*90*_. VOT_90_ is defined as the time period that the fit function needs to reach 90% of the saturation amplitude *r*_sat_ starting from 5% of the saturation amplitude [18] (Figure 5).

### 2.6. Filtered and Unfiltered GAW

**Figure 6:**
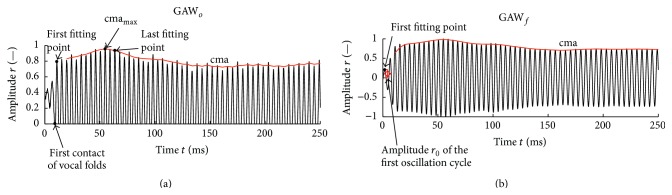
Unfiltered (a) and filtered (b) GAW of the first syllable representing a female subject; central moving average cma, maximum of the central moving average cma_max_, first and last peaks to which the functions are fitted, amplitude *r*_0_ of first oscillation cycle of GAW_*f*_, and first contact of vocal folds are depicted. The fundamental frequency of the signal shown is 231 Hz.

For each GAW, the VOTs were computed for the original unfiltered signal GAW_*o*_ [18] and for the filtered signal GAW_*f*_ according to Mergell et al. [5] (Figure 6).

**Figure 7:**
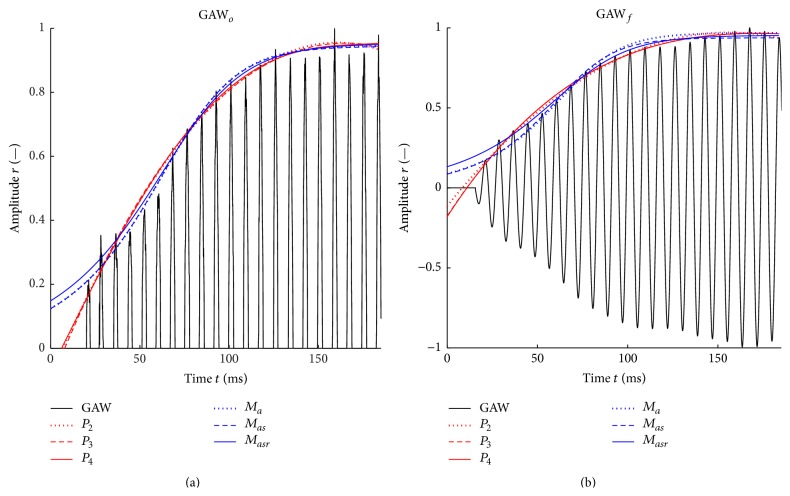
Applied analytical fit functions according to Table 1 approximating the envelope of a male subject's unfiltered (a) and filtered (b) GAW.

The GAWs were filtered using a fourth-order Butterworth band-pass filter reaching from 0.7 to 1.3 times the fundamental frequency *f*_0_ of the respective GAWs. The fundamental frequency *f*_0_ is computed for the first 180 ms of the GAWs. For the male subject group *f*_0_ was between 87 Hz and 131 Hz and for the female subject group *f*_0_ was between 184 Hz and 243 Hz. This filtering frequency band was chosen owing to the relatively strong variation of *f*_0_ at the beginning of the word [‘mama]. The band-pass filter has the following advantages:

(1) The low-pass filter eliminates high frequencies and hence smoothes the GAW. In this way, the actual maximum of each glottal cycle can be determined more accurately. High-frequency artifacts might be generated by user-caused quality variations of the semiautomatic segmentation, which can result in a slightly incorrect glottal area determined for each picture. High-frequency artifacts may also be caused by reduced quality of the hs recordings. The quality of the hs recordings can be affected, for example, by slight movements of the endoscope or reduced image quality caused by foggy endoscope optics.
(2) The high-pass filter removes low frequencies and therefore eliminates the offset so that the fit function (as presented by Mergell et al. [5]) that also naturally converges to zero lim_*t*→−*∞*_⁡*M*(*t*) = 0 (Figure 7) becomes reasonable.

When fitting the unfiltered GAWs, only peaks after the first vocal fold contact were included [18]. For filtered GAWs, all peaks occurring, including those before the first vocal fold contact, were included as fitting points [5] (Figure 6).

### 2.7. Fit Functions and Optimization Methods

Six analytic fit functions were compared (Figure 7, Table 1). They were optimized by a nonlinear least-squares curve fitting within a MATLAB script. Three of the six fit functions are based on the analytical function *M*(*t*) (1) presented in Mergell et al. [5]:

(1) $$\begin{array}{c} M\left(\;t\;\right)=r_{0}\frac{1}{\sqrt{\left(1-x_{i}\right)e^{-2at}+x_{i}}}\;\text{with }x_{i}={\left(\frac{r_{0}}{r_{\mathrm{sat}}}\right)}^{2}. \end{array}$$

Within *M*(*t*), the parameter *a* is the reciprocal of VOT_67_. Therefore, for *M* functions, just the VOT_67_ and not the VOT_90_ is computed. The parameter *r*_0_ is the peak amplitude of the normalized GAW within the first phonation cycle (Figures 5 and 6). In contrast to Mergell et al. [5], we additionally optimized *r*_0_ and *r*_sat_ to increase the degrees of freedom for yielding the least RMS error between the peak amplitudes of all oscillation cycles during onset and the approximated envelope determined by *M*(*t*).

To judge the impact of a single optimization parameters, three functions *M*_*a*_(*t*), *M*_*as*_(*t*), and *M*_*asr*_(*t*) were applied that differ in the number of parameters to be optimized, which are indicated by the subscripts (Table 1, column 2). These optimization parameters were varied within physically reasonable boundaries to accelerate the identification of best fit as indicated in the third column of Table 1.

Moreover, three polynomial fit functions of second, third, and fourth order were tested:

(2) P2t=a0+a1t+a2t2,P3t=a0+a1t+a2t2+a3t3,P4t=a0+a1t+a2t2+a3t3+a4t4.

**Figure 8:**
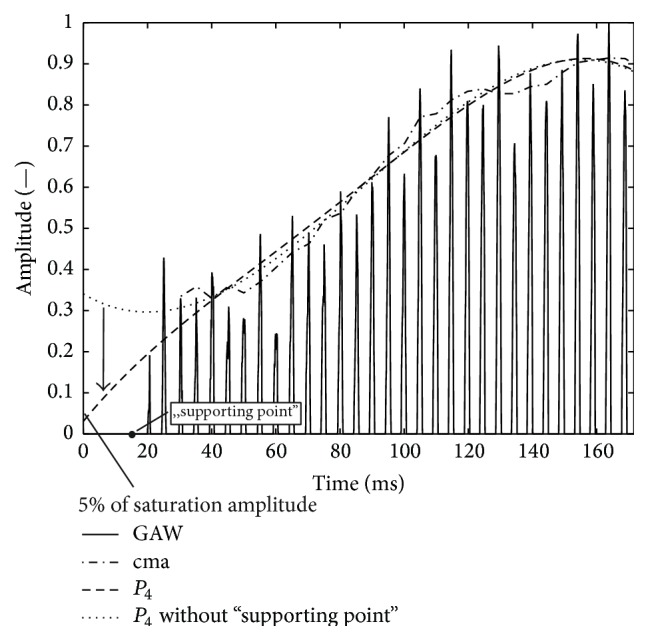
GAW_*o*_ representing a female subject with fourth-order polynomials fitted to peaks; dashed line: polynomial fit function to peaks of GAW; dotted line: polynomial fit function to peaks of GAW plus “supporting point” before GAW. The arrow represents the influence of the additional “supporting point”.

For the polynomial fit functions, the coefficients *a*_*i*_ were optimized to find the best fit of the GAW envelope during phonation onset with a low error. As criterion, the RMSE between the maximum values of the GAW and the fit curve was evaluated. During the optimization process, the ranges of the variations of the coefficients *a*_*i*_ were not restricted to find the global optimum for the approximation of the GAW envelope. An additional “supporting point” at *r* = 0 was added to the polynomial fit functions at the distance of one oscillation cycle before the first fitting point to prevent the polynomial fit functions from rising to *t* → 0 ms. If the polynomial fit function rises to *t* → 0 ms (Figure 8, dotted line), tests have shown that the polynomial fit function does not reach the 5% or 32.2% of the saturation amplitude as shown in Figure 8 for the polynomial fit function *P*_4_ without supporting point (dotted line) and the VOT cannot be determined.

### 2.8. Evaluation of Fit Function Quality

To judge the quality of the different analytic fit functions and to compare the results for unfiltered and filtered GAWs and the male and female groups, the three parameters standard deviation of the VOT, reliability, and root mean square error (RMSE) of the fit functions were investigated:

1. The standard deviation of the voice onset time (VOT) gives information on the* consistency* of the VOT computation. It is desired to have a small standard deviation to be able to separate physiological from pathological VOTs in the future clinical applications. Therefore, the analytic fit functions can be classified in terms of their ability to reproduce physiological VOTs within a narrow time interval.
2. The parameter* reliability* is the percentage of GAWs for which the VOT is computable, that is, if a fit function lies in the defined intervals (VOT_67_: 32.2–67.8%; VOT_90_: 5–90%) related to the saturation amplitude (Section 2.5). It shows how reliably the VOT can be computed by each fit function. Furthermore, a comparison between VOT_90_ and VOT_67_ is performed for the polynomial fit functions.
3. The accuracy of the approximation given by* RMSE* shows the normalized error between the fit function and the peaks of each glottal cycle in the GAW. The RMSE indicates how accurate the fit function is to the GAW.

The suitability of the different analytic fit functions for computing the VOT is finally determined by combining the results for the three parameters standard deviation of VOT, reliability, and RMSE.

## 3. Results and Discussion

### 3.1. Reliability of Fit Functions

#### 3.1.1. VOT_67_

In Table 2, the reliability is listed for the analytic fit functions for the female and male groups and for filtered (GAW_*f*_) and unfiltered (GAW_*o*_) data.

For GAW_*o*_s of the female group, all *M* functions have a reliability of 100%. Within the polynomial fit function group, *P*_4_ and *P*_3_ show the highest reliabilities, with 98%. For GAW_*f*_, a 100% reliability is reached by all *M* fit functions and by *P*_4_. Comparing GAW_*o*_ with GAW_*f*_, all of the *M* fit functions show a reliability of 100% for the GAW_*o*_s and for the GAW_*f*_s. The reliabilities of the polynomial fit functions are higher for the GAW_*f*_s than for the GAW_*o*_s.

For GAW_*o*_s of the male group, *P*_3_, *P*_4_, and *M*_*a*_ reach a reliability of 100%. For GAW_*f*_, *P*_3_, *P*_4_ and *M*_*a*_ reach a reliability of 100%. *P*_2_ has the lowest reliability (97%). Comparing GAW_*o*_ with GAW_*f*_, the highest reliability of 100% is achieved by *P*_3_, *P*_4_, and *M*_*a*_. Whereas *M*_*asr*_ shows a reliability of 99% for both the filtered and the unfiltered data, *M*_*as*_ and *P*_2_ have a 1% higher reliability for GAW_*o*_s than for GAW_*f*_s. In contrast to the female group, the reliabilities of the polynomial fit functions are slightly higher for the GAW_*o*_ compared with GAW_*f*_.

A reliability of 100% in both gender groups and for GAW_*f*_s and GAW_*o*_s is achieved by *M*_*a*_, which is therefore the most robust fit function. *P*_4_ has a reliability of 100% for both the male and female groups for the GAW_*f*_s and of 100% and 98% for the GAW_*o*_s.  *M*_*asr*_ shows a reliability of at least 99%. *M*_*a*_ and *P*_3_ have reliabilities between 100% and 98%. *P*_2_ is the least robust fit function. In summary, for VOT_67_, a similar high reliability is given for all fit functions except *P*_2_.

#### 3.1.2. VOT_90_

In Table 3, the reliability for the analytic fit functions *P*_2_^90^, *P*_3_^90^, and *P*_4_^90^ is listed for the female and male groups and for filtered and unfiltered data. In general, the reliability is lower than for VOT_67_ owing to the stricter definition of the VOT_90_.

For the female group, *P*_4_^90^ shows the highest reliability for GAW_*o*_ and GAW_*f*_, with 94% and 93%. Basically, the reliability increases with the order of the polynomial. For the male group, the highest reliability for GAW_*o*_ and GAW_*f*_ is shown by *P*_4_^90^, with 98%. Again, the reliability increases with the order of the polynomial. Concerning both gender groups, *P*_4_^90^ shows the highest reliability for the unfiltered and filtered GAW.

#### 3.1.3. Onset Time Definition VOT_67_ versus VOT_90_

The comparison of the reliability between the fit functions for the two onset time definitions VOT_67_ and VOT_90_ reveals better results for VOT_67_. However, higher reliabilities for VOT_67_ were expected, since by definition functions reaching 5% of the saturation amplitudes naturally always reach 32.2% of the saturation amplitude (Figure 8). Regarding reliability, the best combination would be VOT_67_ in combination with any fit function except *P*_2_.

### 3.2. Consistency of Fit Functions

#### 3.2.1. VOT_67_

In Table 4, mean VOT_67_s and their standard deviations (SDs) are listed. For both gender groups,* M* functions have significantly higher VOT_67_s and SDs than polynomial fit functions for GAW_*f*_ and GAW_*o*_. VOT_67_s of the *M* functions basically increase with the number of optimized parameters. For the polynomial fit functions, VOT_67_s decrease with the polynomial degree (51 → 32 ms). For the polynomial fit functions, the SDs of VOT_67_s are in similar range between 21 and 37 ms (Δ(*t*) = 16 ms). In contrast, the SDs of the *M* functions spread over an interval from 71 to 241 ms (Δ(*t*) = 170 ms).

Overall, the most consistent combination for both gender groups is given by *P*_2_, *P*_3_, and *P*_4_ for unfiltered data (21 ms ≤ SD ≤ 34 ms); however, *P*_2_, *P*_3_, and *P*_4_ show almost identical consistent results for the filtered data (28 ms ≤ SD ≤ 37 ms).

#### 3.2.2. VOT_90_

In Table 5, mean VOT_90_s and their standard deviations (SDs) are listed for the analytic fit functions *P*_2_^90^, *P*_3_^90^, and *P*_4_^90^ for the female and male groups and for filtered and unfiltered data. For both gender groups, the polynomial fit functions show similar consistency for filtered and unfiltered GAW (57 ms ≤ SD ≤ 63 ms). Also, the absolute VOT values are very similar (88 ms ≤ VOT_90_ ≤ 116 ms). Overall, the most consistent combination for both gender groups is given by *P*_2_^90^ and *P*_4_^90^ owing to the smaller standard deviation than for *P*_3_^90^. Hence computing VOT_90_ for GAW_*f*_ or GAW_*o*_ yields equally good results.

#### 3.2.3. Comparison of VOT_90_ with VOT_67_

Owing to the definitions of VOT_90_ and VOT_67_, it was expected that VOT_90_ values would be significantly higher than VOT_67_ values. However, regarding the consistency, it was not obvious. Here, the SD values for VOT_67_ were only about half (≤34 ms) those for VOT_90_ (≥56 ms). Hence, from the consistency point of view, the best choice would be a polynomial fit function (*P*_2_, *P*_3_, *P*_4_) for filtered or unfiltered GAW applying VOT_67_.

### 3.3. Accuracy: Root Mean Square Error (RMSE)

In Table 6, the RMSEs of all fit functions and their SDs are given on the basis of the experimental data for which VOT_67_ could be reliably computed. For the computation of the RMSE, all peaks of the GAW that contributed to the determination of the corresponding fit function were used.

All values for the *M* functions are given only for VOT_67_. For the polynomial fit functions, the RMSEs are equal on comparing VOT_90_ and VOT_67_ since these two definitions differ only in the time considered to compute the onset time interval.

Regarding *M* functions, the best performance for both gender groups and for filtered and unfiltered GAW is provided by *M*_*asr*_ (0.07 ≤ RMSE ≤ 0.08). Of the polynomial fit functions, *P*_3_ and *P*_4_ show the best performance (0.06 ≤ RMSE ≤ 0.08). In summary, *P*_4_ for the GAW_*f*_s has the lowest RMSE values in addition to very small SDs, followed by *P*_4_ (GAW_*o*_) and *M*_*asr*_. The highest RMSEs are shown by *M*_*a*_.

### 3.4. Evaluation of the Applied Methods

For the three different criteria, the following best combinations were found:

(i) *Reliability*: VOT_67_ combination with any fit function except *P*_2_.
(ii) *Consistency*: polynomial fit functions (*P*_2_, *P*_3_, *P*_4_) for filtered and unfiltered GAW applying VOT_67_.
(iii) *Accuracy (RMSE)*: *P*_4_ for the GAW_*f*_ independent of VOT definition.

Combining the results for the three criteria yields the conclusion that the most reliable way to compute the VOT is to fit a fourth-order polynomial fit function (*P*_4_) to the filtered glottis area waveform (GAW_*f*_) with the onset time definition VOT_67_ (onset time equals the time between 32.2 and 67.8% of the saturation state). A highly important advantage of VOT_67_ is the high consistency (i.e., small SD of onset time) compared with VOT_90_. This is very important with regard to potential future clinical use, since the physiological onset time of the norm group can only be differentiated from the pathological onset time if the pathological VOT lies outside the given norm onset time interval.

## 4. Conclusion

A study on computing VOT based on GAW from the word [‘mama] derived from HSE data obtained with a flexible endoscope was conducted in order to determine the most robust and reliable method. Different-order polynomial fit functions and a Hopf bifurcation function were tested that approximate the transient VO process, exhibiting increasing amplitudes of the glottal area with a different number of parameters being optimized. The results for filtered and unfiltered GAWs were compared. As a measure of the suitability of a method, a combination of the three different criteria was chosen: the reliability, that is, the percentage of GAWs for which the VOT was computable, VOT itself and its SD, and the RMSE between the fit function and the peak values of the GAW. In summary, the results suggest applying a fourth-order polynomial to approximate the voice onset by fitting it to peak values of the GAW. Furthermore, preprocessing of the GAW in the form of a band-pass filter around *f*_0_ in combination with the VOT_67_ (period between 32.2 and 67.8% of the saturation amplitude of the first syllable of the word [‘mama]) is most advantageous regarding reliability and consistency.

Analyzing the VO based on a GAW derived from HSE data obtained with a flexible endoscope combines different benefits. The vocal fold dynamics reflect the actual basic voice signal which is not modified by the vocal tract as the acoustic voice signal. The flexible endoscope affects the voice production less than a rigid endoscope and allows recording of articulations. Moreover, the GAW reflects the entire vocal fold dynamics, in contrast to trajectories which reflect the vocal fold dynamics just at one thin line. However, owing to image quality, not all of the HSE results could be included in the VOT computation. With a new generation of hs cameras, image quality in terms of brightness, contrast, and spatial resolution will be increased [9]. Moreover, the temporal resolution of the hs recordings can be increased, so that at some point it will keep up with acoustic voice recordings.

As perspective, the data in this paper are a contribution to the establishment of VOT as clinical measure in clinical diagnostics and therapy for voice disorders, especially functional dysphonia. The results of the reliability, robustness, and accuracy of the different methodologies for the determination of the VOT are suited to contribute to the standardization of this measure.

## Figures and Tables

**Table 1:** Applied analytic fit functions *M*(*t*), *P*_*i*_(*t*), and *P*_*i*_^90^(*t*), optimization parameters, and optimization boundaries.

Analytic fit functions	Optimization parameters	Optimization boundaries
*M*_*a*_	*a*/(1/s)	[11000]

*M*_*as*_	*a*/(—)	[11000],
	*r*_sat⁡_/(—)	[02]

*M*_*asr*_	*a*/(1/s)	[11000],
	*r*_sat⁡_/(—)	[02],
	*r*_0_/(—)	[01]

*P*_2_, *P*_2_^90^	*a*_0_, *a*_1_, *a*_2_	—

*P*_3_, *P*_3_^90^	*a*_0_, *a*_1_, *a*_2_, *a*_3_	—

*P*_4_, *P*_4_^90^	*a*_0_, *a*_1_, *a*_2_, *a*_3_, *a*_4_	—

**Table 2:** Reliabilities (%) for the six fit functions for both gender groups for GAW_*o*_s and GAW_*f*_s. All fit functions achieve high values of at least 94%.

Reliability (%)	*M*_*a*_	*M*_*as*_	*M*_*asr*_	*P*_2_	*P*_3_	*P*_4_
Female group						
GAW_*o*_	100	100	100	94	98	98
GAW_*f*_	100	100	100	96	99	100

Male group						
GAW_*o*_	100	99	99	98	100	100
GAW_*f*_	100	98	99	97	100	100

**Table 3:** Reliabilities for the polynomial fit functions for both gender groups for GAW_*o*_s and GAW_*f*_s.

Reliabilites (%)	*P*_2_^90^	*P*_3_^90^	*P*_4_^90^
Female group			
GAW_*o*_	85	89	94
GAW_*f*_	90	91	93

Male group			
GAW_*o*_	94	96	98
GAW_*f*_	95	94	98

**Table 4:** Mean values for VOT_67_ and the standard deviation (SD) listed for all fit functions and both gender groups.

VOT_67_ (ms)	*M*_*a*_	*M*_*as*_	*M*_*asr*_	*P*_2_	*P*_3_	*P*_4_
Female group						
GAW_*o*_	118	144	152	51	41	37
SD	220	241	233	33	34	34
GAW_*f*_	32	29	66	47	41	39
SD	28	31	106	30	35	36

Male group						
GAW_*o*_	58	94	97	43	33	32
SD	109	200	189	29	21	25
GAW_*f*_	71	82	117	41	35	34
SD	179	209	238	28	32	37

**Table 5:** Mean values for VOT_90_ (ms) and the standard deviation (SD) for *P*_2_^90^, *P*_3_^90^, and *P*_4_^90^.

VOT_90_ (ms)	*P*_2_^90^	*P*_3_^90^	*P*_4_^90^
Female group			
GAW_*o*_	116	113	102
SD	59	73	62
GAW_*f*_	110	110	103
SD	57	69	59

Male group			
GAW_*o*_	103	94	92
SD	59	60	59
GAW_*f*_	96	94	88
SD	56	71	63

**Table 6:** Mean RMSEs and SDs of the applied fit functions for filtered and unfiltered GAW and both gender groups.

RMSE	*M*_*a*_	*M*_*as*_	*M*_*asr*_	*P*_2_	*P*_3_	*P*_4_
Female group						
GAW_*o*_	0.09	0.08	0.07	0.08	0.08	0.07
SD	0.06	0.05	0.04	0.03	0.03	0.03
GAW_*f*_	0.11	0.09	0.07	0.08	0.7	0.06
SD	0.05	0.04	0.03	0.03	0.03	0.03

Male group						
GAW_*o*_	0.11	0.09	0.08	0.10	0.08	0.07
SD	0.06	0.05	0.04	0.04	0.03	0.03
GAW_*f*_	0.10	0.09	0.08	0.10	0.08	0.07
SD	0.06	0.05	0.05	0.06	0.06	0.05
